# Revising a Personal Genome by Comparing and Combining Data from Two Different Sequencing Platforms

**DOI:** 10.1371/journal.pone.0060585

**Published:** 2013-04-08

**Authors:** Deokhoon Kim, Woo-Yeon Kim, Sun-Young Lee, Sung-Yeoun Lee, Hongseok Yun, Soo-Yong Shin, Jungyoun Lee, Yoojin Hong, Youngmi Won, Seong-Jin Kim, Yong Seok Lee, Sung-Min Ahn

**Affiliations:** 1 Lee Gil Ya Cancer and Diabetes Institute, Gachon University, Incheon, Korea; 2 Bioinformatics Team, Samsung SDS, Seoul, Korea; 3 Department of Translational Medicine, Gachon University Gil Hospital, Incheon, Korea; 4 CHA Cancer Institute, CHA University of Medicine and Science, Seoul, Korea; Leuven University, Belgium

## Abstract

For the robust practice of genomic medicine, sequencing results must be compatible, regardless of the sequencing technologies and algorithms used. Presently, genome sequencing is still an imprecise science and is complicated by differences in the chemistry, coverage, alignment, and variant-calling algorithms. We identified ∼3.33 million single nucleotide variants (SNVs) and ∼3.62 million SNVs in the SJK genome using SOLiD and Illumina data, respectively. Approximately 3 million SNVs were concordant between the two platforms while 68,532 SNVs were discordant; 219,616 SNVs were SOLiD-specific and 516,080 SNVs were Illumina-specific (*i*.*e*., platform-specific). Concordant, discordant, and platform-specific SNVs were further analyzed and characterized. Overall, a large portion of heterozygous SNVs that were discordant with genotyping calls of single nucleotide polymorphism chips were highly confident. Approximately 70% of the platform-specific SNVs were located in regions containing repetitive sequences. Such platform-specificity may arise from differences between platforms, with regard to read length (36 bp and 72 bp vs. 50 bp), insert size (∼100–300 bp vs. ∼1–2 kb), sequencing chemistry (sequencing-by-synthesis using single nucleotides vs. ligation-based sequencing using oligomers), and sequencing quality. When data from the two platforms were merged for variant calling, the proportion of callable regions of the reference genome increased to 99.66%, which was 1.43% higher than the average callability of the two platforms, representing ∼40 million bases. In this study, we compared the differences in sequencing results between two sequencing platforms. Approximately 90% of the SNVs were concordant between the two platforms, yet ∼10% of the SNVs were either discordant or platform-specific, indicating that each platform had its own strengths and weaknesses. When data from the two platforms were merged, both the overall callability of the reference genome and the overall accuracy of the SNVs improved, demonstrating the likelihood that a re-sequenced genome can be revised using complementary data.

## Introduction

Next generation sequencing (NGS) technology has enabled personal genomics through a reduction in costs and an increase in efficiency [1], [2]. After a lag phase in which only a small number of personal genomes were sequenced, we are entering the exponential phase of personal genomics, sequencing thousands of genomes at the population level [3]–[18]. Furthermore, efforts have been made to incorporate personal genome information in clinical assessments [19]. Presently, genome sequencing is still an imprecise science and is complicated by differences in the chemistry, coverage, alignment, and variant-calling algorithms [20].

Single nucleotide variants (SNVs), short insertions/deletions (indels), structural variants (SVs), new sequences, and phasing are unique parameters of genome re-sequencing [21]. The number of total SNVs identified in personal genomes varies significantly from ∼3 to 4 million [3], [8], [11], [16], [17]. For the robust practice of genomic medicine, sequencing results must be compatible, regardless of the sequencing technologies and algorithms used. However, in the study by Bentley et al. [4], the number of total single nucleotide polymorphisms (SNPs) from the same data set varied substantially, depending on the algorithms used. When the same genome was analyzed using another method of sequencing, the results differed, including a different amount of total SNPs acquired [11]. These findings indicated that a rigorous comparison of separate sequencing methods was needed to delineate their limitations, and personal genomes may need to be revised using a complementary data set until a standardized sequencing protocol for medical genomics is established.

Previously, we sequenced the first Korean human genome using the DNA polymerase-based Illumina GA II platform (36 bp and 72 bp in read length; paired-end libraries with insert sizes up to 300 bp; ∼29× sequencing coverage). In this study, we re-sequenced the same Korean individual genome using a different NGS platform (the ABI ligase-based SOLiD platform). The Illumina and SOLiD platforms have two major differences that might potentially affect their outcomes. First, the Illumina platform uses sequencing-by-synthesis chemistry, based on a combination of fluorescent-labeled nucleotides and DNA polymerase [22]. The SOLiD platform uses a ligation-based sequencing method, based on the combination of fluorescent-labeled oligomers and DNA ligase [23]. Second, the Illumina platform mainly uses paired-end libraries and the average fragment size ranges from 100 to 300 bp. The SOLiD platform mainly uses mate-pair libraries and the average fragment size ranges from 1 to 2 kb. Here, we examined potential differences between the two sequencing platforms and combined data to revise the personal genome sequence.

## Materials and Methods

### Library Construction and Sequencing

All study protocols were approved by the Institutional Review Board of Lee Gil Ya Cancer and Diabetes Institute of Gachon University (Approval # GU0911–001).

Genomic DNA (gDNA) was extracted from whole blood with a QIAamp DNA Blood Maxi Kit according to the manufacturer’s instructions (QIAGEN).

Libraries were prepared according to the “SOLiD System Mate-paired Library Preparation” protocol from the SOLiD System: Library Preparation Guide (02/2009 edition).

Briefly, gDNA was fragmented by HydroShear (Genomic Solutions) at the proper settings for targeted sizes. QIAquick Gel Extraction Kit (QIAGEN) was used for subsequent purifications of sheared DNA, enzymatic reactions, and size-selected DNA from agarose gels. To repair damaged DNA ends and obtain 5′-phosphorylated blunt-ends (5′P), the fragments were end-repaired using the End-It DNA End-Repair Kit (Epicentre Biotechnologies). Ligations for adaptor attachment and circularization were accomplished using the Quick Ligation Kit (New England BioLabs). DNA was quantified using a NanoDrop ND 1000 Spectrophotometer (Thermo Fisher Scientific) and Qubit IT dsDNA HS (Invitrogen).

In sequential order, the sheared gDNA fragments were end-repaired; then the LMP CAP Adaptors (missing a 5'-P from one of its oligonucleotides) resulted in a nick on each strand when the DNA was circularized in a later step and were ligated to the end-repaired DNA fragments. The adaptor ligated products were separated on a 1% agarose gel and excised from the gel at approximate positions for span size ranges (1–2 kb). Size-selected DNA fragments were circularized with a biotinylated internal adaptor. Uncircularized DNA fragments were eliminated using Plasmid-Safe ATP-Dependant DNase (Epicentre Biotechnologies). Using the circularized DNA fragments, nick-translation was performed for 14 minutes at 0°C in an ice water bath using DNA polymerase I from *Escherichia coli*. The nick-translated products were cleaved at the nicks using T7 exonuclease and S1 nuclease, and end-repaired as described above. P1 and P2 adaptors, which were used for library amplification, electronic polymerase chain reaction (ePCR), and ligation sequencing, were ligated to the ends of the end-repaired DNA. The ligated DNA then underwent nick translation using DNA polymerase I. The completed library was amplified with Cloned Pfu DNA Polymerase (Stratagene) in eight cycles. The amplified library was separated on a 4% agarose gel and the correct-sized band (275–300 bp) was excised, eluted, and quantified using Qubit IT (Invitrogen).

The templated bead preparation and sequencing steps with SOLiD 3.5 were performed according to the manufacturer’s instructions (Applied Biosystems).

The concentration of each library for ePCR was designed to range between 1.5 and 2.0 pM. Templated beads were deposited onto two slides of full-scale per library, and sequencing was carried out to 50 bp using SOLiD v3 plus chemistry.

### Mapping and Variation Detection

The human reference sequence (version hg18) was obtained from the UCSC Genome Browser database [24]. Using BioScope version 1.2 (http://solidsoftwaretools.com), sequence reads from SOLiD system were aligned to the reference genome. Sequence reads from Illumina GA II system were aligned to the reference genome using BWA with default settings. Aligned data in BAM format were realigned, de-duplicated, and recalibrated. For variant-calling, UnifiedGenotyper walker in GATK (v 1.5–12) was used. To estimate the overall accuracy of data, SNVs identified in sequencing data were compared with genotyping data from SNP chip (Affymetrix 6.0) [3]. The SNVs were evaluated by comparison with SNP chip data while incorporating reference genome information.

### Detection of Callable and Non-callable Regions

The CallableLoci walker in GATK was used with default settings to detect callable regions of the reference genome using aligned data. The thresholds of callability used by CallableLoci walker were a minimum sequencing depth equal to 4 and a minimum mapping quality equal to 10.

## Results

### Sequencing and Mapping with Short Reads

Previously, we sequenced the SJK genome using the DNA polymerase-based Illumina GA II platform with paired-end sequencing [3]. In this study, we sequenced the same genome using the DNA ligase-based SOLiD platform to compare and combine the sequencing data from two separate NGS platforms [11]. We generated 178.29 gigabases (Gb) of ∼3.0 billion mate-paired/single-end reads with a read length of 50 base pairs (bp). Approximately 2.9 billion reads were aligned to the reference human genome (hg18) using Bioscope version 1.2.

### Identification and Comparative Analysis of SNVs

**Figure 1:**
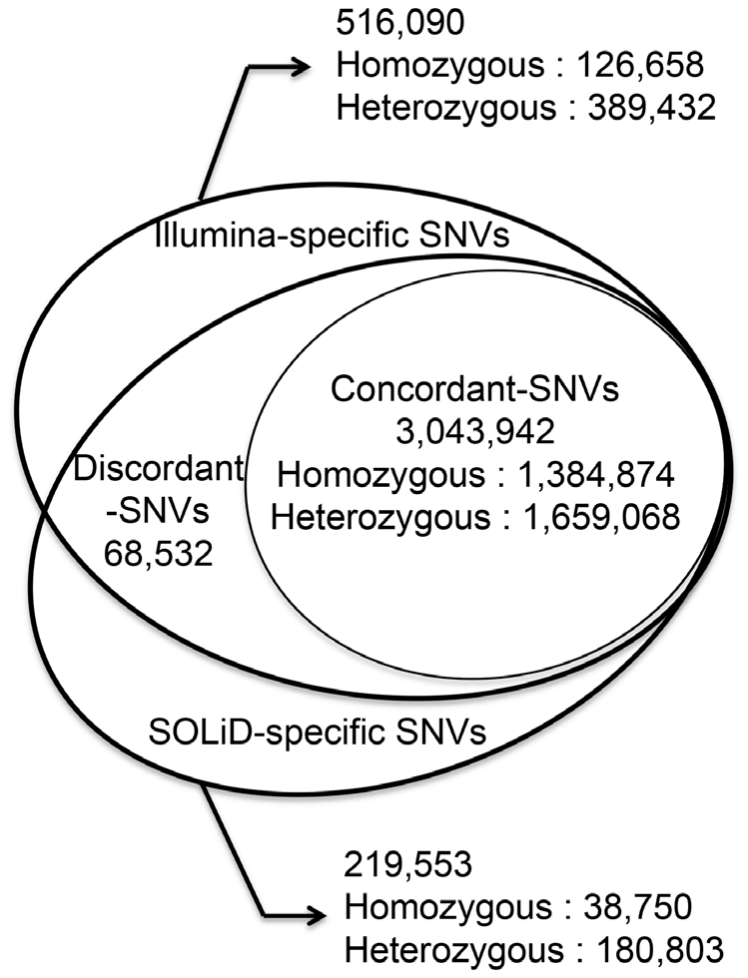
Concordance of SNVs identified by the two different sequencing platforms.

For data comparison, GATK version 1.5, which can be used for both Illumina and SOLiD platforms, was applied to call variants [25]. Using GATK, we identified ∼3.33 million SNVs in the SJK genome using SOLiD data and ∼3.62 million SNVs using Illumina data. Figure 1 summarizes the comparative analysis of SNVs identified by these two platforms. Approximately 3 million SNVs were concordant between the platforms while 68,532 SNVs were discordant; 219,616 SNVs were SOLiD-specific and 516,080 SNVs were Illumina-specific (*i*.*e*., platform-specific).

To assess the overall accuracy of the SNVs in each group (*i*.*e*., concordant, discordant, or platform-specific), we compared the SNVs with genotyping results from Affymetrix SNP 6.0 chip (chip-concordance rate) (Table 1). The chip-concordance rate of concordant SNVs was 98.92%; those of discordant SNVs from SOLiD and Illumina data were 9.81% and 90.08%, respectively, while those of SOLiD-specific and Illumina-specific SNVs were 97.11% and 97.82%, respectively.

**Table 1:** Classification of sequenced SNVs and their chip-concordance.

			Illumina			SOLiD		
			# of SNVs	Chip-concordance	Median depth	# of SNVs	Chip-concordance	Median depth
		Total	390,494	98.92%	20	390,494	98.92%	35
	Chip-concordant	HOM	192,914	48.87%	20	192.914	48.87%	32
Concordant SNVs between platforms		HET	197,580	50.05%	20	197,580	50.05%	38
		Total	4,244	–	21	4,244	–	37
	Chip-discordant	HOM	564	–	19	564	–	29
		HET	3,680	–	21	3,680	–	38
		Total	2,489	90.08%	18	271	9.81%	29
	Chip-concordant	HOM	2,440	88.31%	17	127	4.60%	31
Discordant SNVs between platforms		HET	49	1.77%	18	144	5.21%	27
		Total	274	–	13	2,492	–	21
	Chip-discordant	HOM	144	–	12	50	–	10.5
		HET	130	–	13	2,442	–	21
Illumina-specific SNVs	Chip-concordant	Total	5,879	97.82%	18	–	–	–
	Chip-discordant	Total	131	–	20	–	–	–
SOLiD-specific SNVs	Chip-concordant	Total	-	–	–	3,565	97.11%	33
	Chip-discordant	Total	-	–	–	106	–	27

HOM, homozygous calls; HET, heterozygous calls; Median depth, median sequencing depth

### Concordant SNVs between Platforms

The chip-concordance rate of concordant SNVs was 98.92%. Table 2 summarizes the patterns of chip-discordance. The majority of chip-discordant calls from sequencing data were heterozygous, whereas the majority of chip-discordant calls from chip data were homozygous, and vice versa. To assess the accuracy of heterozygous SNVs from the sequencing data, which were chip-discordant, we calculated the sequencing depths and percentages of each base in the heterozygous SNVs. The criteria for highly confident heterozygous calls using SOLiD were as follows: more than 20× coverage, and the second most frequent base had to be >30%. Out of 3,677 heterozygous SNVs that were chip-discordant, 2,923 SNVs (80%) met these stringent criteria (Table S1). This finding indicated that chip data may be prone to a heterozygous-to-homozygous error in these regions.

**Table 2:** Patterns of chip-discordance in concordant SNVs between platforms.

		Sequencing	
		Homozygous	Heterozygous
Chip	Homozygous	71 (1.67%)	3,677 (86.64%)
	Heterozygous	493 (11.62%)	3 (0.07%)

### Discordant SNVs between Platforms

The chip-concordance rate of discordant SNVs was substantially lower than those of concordant or platform-specific SNVs. As summarized in Table 1, the key feature of this group was that most of the SNVs were homozygous in Illumina data, but heterozygous in SOLiD data. The chip-concordance rate of homozygous SNVs in Illumina data was 88.31%, while that of heterozygous SNVs in SOLiD data was 4.60%. Specifically, the majority of SNVs in this group were homozygous in Illumina data and heterozygous in SOLiD data. Genome re-sequencing was reported as being prone to heterozygous-to-homozygous sequencing errors because additional sequencing depth is required to accurately call heterozygous variants [26]. Additionally, since the chip data may contain heterozygous-to-homozygous errors, chip-concordance cannot be used to estimate the overall accuracy of data since the majority of discordance between the two platforms comes from homozygous calls in one platform and heterozygous calls in the other platform.

**Figure 2:**
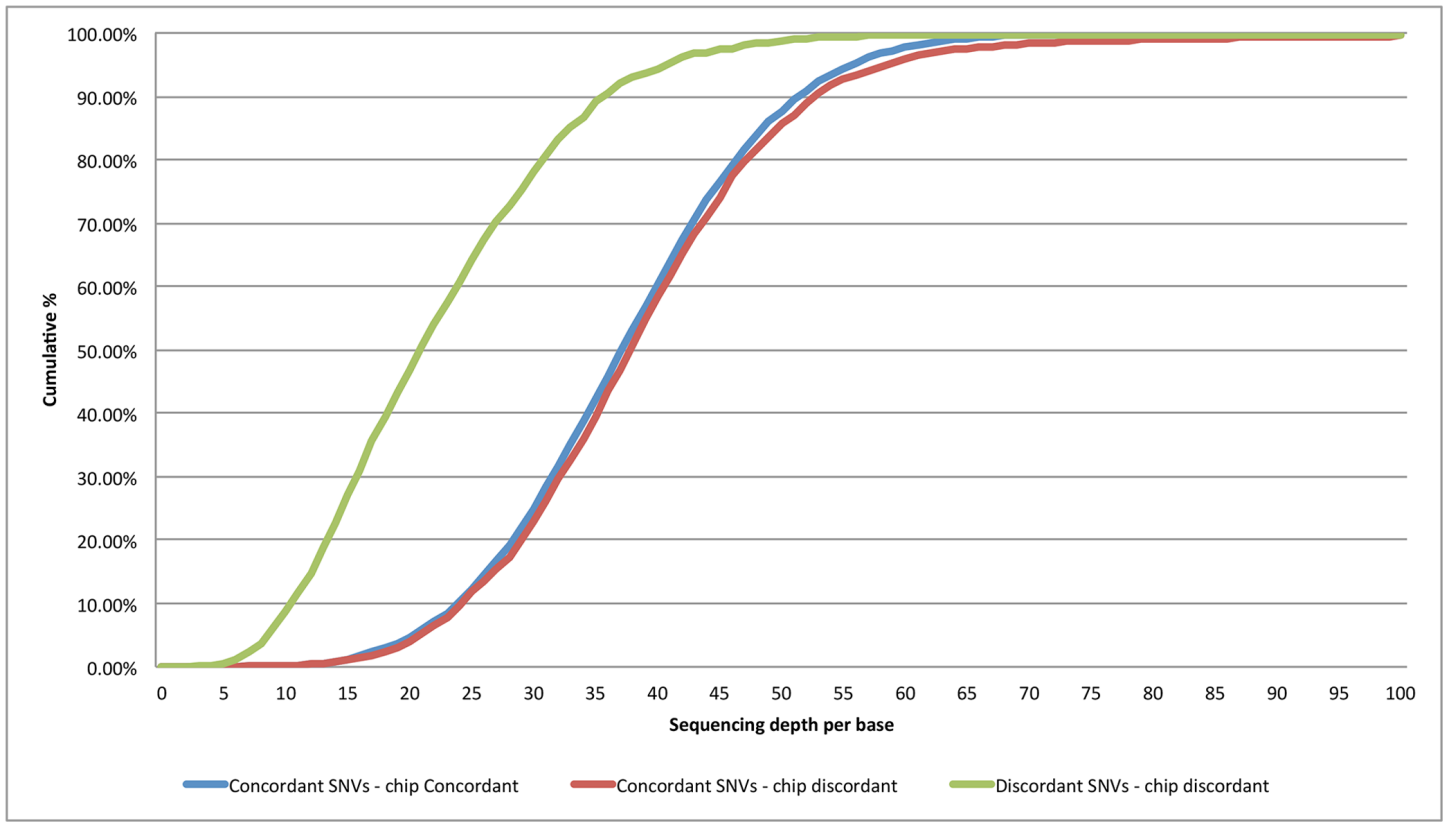
Cumulative frequency plot of sequencing depths in heterozygous calls. The sequencing depths of heterozygous calls in the SOLiD data are plotted. The patterns of concordant SNVs that are either chip-concordant or chip-discordant are almost compatible, which explains why the majority of heterozygous concordant SNVs that are chip-concordant are highly confident calls. In contrast, the median of discordant SNVs that are chip-discordant is substantially lower than those of concordant SNVs, which explains why only 25% of them are highly confident calls.

To better understand this discrepancy of discordant SNVs between platforms, we applied the same stringent criteria for highly confident heterozygous calls to the SOLiD data. Compared to the chip-discordant group, only 25% of the heterozygous concordant SNVs among the two platforms met the criteria, 80% of which were highly confident calls (*i*.*e*., over 20× sequencing depth and the second most frequent base was over 30%). To determine the cause of this difference, we compared the sequencing depths of heterozygous calls in each group. As illustrated in Figure 2, the sequencing depths of heterozygous SNVs differed significantly between the concordant and discordant SNVs. The median sequencing depth of heterozygous SNVs concordant between platforms and chip-concordant was 38, while that of heterozygous SNVs concordant between platforms and chip-discordant was 38 and that of heterozygous SNVs discordant between platforms and chip-discordant was only 21. In this group, 25% or more of the SNVs that were called heterozygous were highly confident calls. However, the fidelity of data decreased in the rest of the SNVs due to low sequencing depth.

### Platform-specific SNVs

As illustrated in Figure 1, platform-specific SNVs were detected in one of the two platforms (*i*.*e*., 516,090 SNVs from Illumina data and 219,533 SNVs from SOLiD data). In comparison to the overall heterozygosity rate of ∼60% in the SJK genome, the heterozygosity rates of these SNVs were relatively high (75.46% in Illumina data and 82.35% in SOLiD data). When DNA was screened using the RepeatMasker database, 71.36% of the Illumina-specific SNVs and 70.92% of the SOLiD-specific SNVs were located in repetitive regions. The chip-concordance rates of platform-specific SNVs were 97.82% in Illumina data and 97.11% in SOLiD data, which were relatively high, and indicate that the overall accuracy is reliable. This platform-specificity in sequencing data may arise from differences between platforms with regard to read lengths (36 bp and 72 bp vs. 50 bp), insert sizes (∼100–300 bp vs. ∼1–2 kb), sequencing chemistry (sequencing-by-synthesis using single nucleotides vs. ligation-based sequencing using oligomers), and sequencing quality.

### Callable and Non-callable Regions of the Genome

When a genome is re-sequenced against a reference genome, not every sequence of the reference genome is re-sequenced. Specifically, after genome re-sequencing, callable and non-callable regions of the genome are present, depending on the results [27].

**Figure 3:**
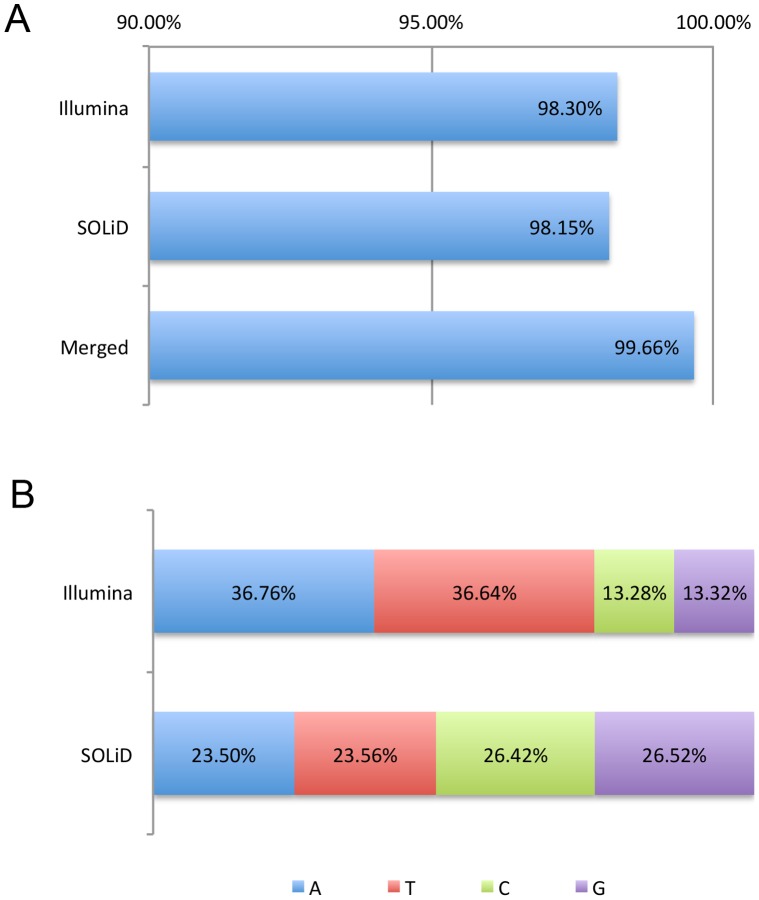
Callable and non-callable regions. (A) Using Illumina and SOLiD data, 98.3% and 98.15% of the reference genome are callable, respectively. Using the merged data, the callability increases to 99.66%, which is 1.43% higher than the average callability of two platforms, representing about 40 million bases. (B) The base composition of non-callable regions. In SOLiD data, the proportions of A, T, C, and G were almost even. In Illumina data, the proportions of A and T were higher than those of C and G.

Using the CallableLoci walker in GATK, which estimates the callability based on both sequencing depth and mapping quality, we categorized each base into callable, poor mapping quality, low coverage, and no coverage groups. In Illumina and SOLiD platforms, callable and poor mapping quality bases cover 98.30% and 98.15% of the reference genome, respectively (Figure 3A). When the base composition of non-callable regions was analyzed, the two platforms showed slightly different patterns. The base composition of non-callable regions in SOLiD data was similar, while the proportions of A and T were twice higher than those of C and G in Illumina data (Figure 3B).

To increase the callability of the reference genome by combining the sequencing data from the platforms, we merged the sequencing data using BAM files, and called variants using GATK, and again categorized each base into callable, poor mapping quality, low coverage, and no coverage groups. As illustrated in Figure 3B, the proportion of callable regions in the merged data was 99.66%, which was 1.43% higher than the average callability of the two platforms, representing ∼40 million bases (Figure 3A).

To estimate the overall accuracy of data in each group, we calculated the chip-concordance. As summarized in Table 3, the chip-concordance of callable regions in merged data was 99.26, which was 0.7% higher than the average chip-concordance of callable regions in Illumina and SOLiD. We performed the chi-square test and the result was statistically significant (*P*<0.001, odds ratio [OR] = 1.53 [merged vs. Illumina] and 2.11 [merged vs. SOLiD]). This demonstrates that the overall accuracy, as well as the overall callability, improved in the merged data upon combining data from the two platforms.

**Table 3:** Chip-concordance of callable and non-callable regions.

	Chip-concordance	
	Callable regions	Non-callable regions
Illumina data	99.26%	0%
SOLiD data	98.76%	0.14%
Merged data	98.36%	0.04%

## Discussion

In this study, we compared the differences in sequencing results from two sequencing platforms. Approximately 90% of the SNVs were concordant between the platforms, and yet ∼10% of the SNVs were either discordant or platform-specific, indicating that each platform had its own strengths and weaknesses. When data from the two platforms were merged, both the overall callability of the reference genome and the overall accuracy of the SNVs improved, demonstrating that a re-sequenced genome can be revised using complementary data.

### Concordance and Discordance between the Two Platforms

As summarized in Figure 1, SNVs were categorized into concordant, discordant, and platform-specific groups, depending on their concordance between the platforms. To estimate the overall accuracy of the SNVs sequenced, we used their concordance rate and SNP chip genotyping data, a common re-sequencing practice. In the concordant group of SNVs between the platforms, the majority of chip-discordant SNVs were heterozygous in sequencing data but homozygous in chip data. In the discordant group of SNVs between the platforms, the majority of discordant SNVs were heterozygous in one platform and homozygous in the other platform. Specifically, the majority of discordance between genotyping platforms, either between genotyping chip and sequencing platforms, or between sequencing platforms, were homozygous-to-heterozygous (or vice versa) errors rather than homozygous-to-homozygous or heterozygous-to-heterozygous. Heterozygous-to-homozygous errors are mainly due to sequencing depths in sequencing data. Paradoxically, this may imply that heterozygous calls from sequencing data, especially those that meet highly stringent criteria, can be regarded as confident calls. Evidence supporting this argument is that the majority of chip-discordant SNVs that were concordant between platforms were heterozygous in sequencing data but homozygous in chip data. Approximately 80% of these SNVs in sequencing data were highly confident heterozygous calls.

### Alignment of Sequencing Data

When we compare the sequencing data generated by two different platforms, the best way would be to use the same downstream bioinformatics pipeline (e.g., mapping and variant calling). Several aligners can handle data from both the Illumina and SOLiD platforms, including BWA [28], BOWTIE [29], and BFAST [30]. When we tested them, however, the mapping results were very different; furthermore, BWA is rarely used for SOLiD data, and BFAST is rarely used for Illumina data.

Therefore, we used the most commonly used aligners for each platform. Default parameters were also used in the same context. In the literature, BWA and Bioscope were often used with the default parameters for alignment [31]–[34].

According to the concordance analysis with SNP chip data, the performance of data from each platform with the current alignment is within the acceptable reported range [35]. Therefore, we assumed that the choice of the most commonly used aligner for each platform would not seriously affect the aim of our study.

In addition, we used hg18 as the reference genome for aligning the sequencing data. There are two reasons why we used hg18 instead of hg19. First, in our previous study of the SJK genome [36], we used hg18 as the reference genome (i.e., much background work that was not included in the previous publication was done). We wanted to build on what we have already done. Second, hg18 is still broadly used, as we can see in recent publications, because hg18 has more UCSC annotations [37]–[41].

### Clinical Perspectives

For medical purposes, the accuracy of sequencing data is very important. Although a futuristic scenario of personal genomics would be whole-genome sequencing followed by continuous interpretation and annotation throughout a lifetime for personalized medicine, accurately sequencing a personal genome remains a challenge. Although a more systematic comparison is required, we showed that comparing and combining sequencing data from two sequencing platforms might allow one to revise a personal genome to a meaningful extent. This approach, however, increases the overall cost of personal genome sequencing, which will be a major limiting factor in its implementation. An alternative approach to this problem is to define the regions of the genome for medical purposes and to characterize non-callable or poorly callable regions using each sequencing platform. For example, we revealed that the majority of platform-specific SNVs were located in regions containing repetitive sequences. Without a specific purpose (e.g., identification of repeat expansions that are associated with certain diseases), we may not need to revise a personal genome for SNVs. Also, with an increasing amount of personal genomics data being released, we may be able to identify regions of the reference genome that are either non-callable or poorly callable using a certain sequencing platform. Then, we may able to target those regions specifically for medicinal purposes using cost-effective complementary sequencing methods.

## Supporting Information

Table S1 **Patterns of base calls in highly confident heterozygous SNVs that are concordant between platforms.**
(XLSX)
